# Setting Priorities for Optimizing Vascular Access Decision Making – An International Survey of Patients and Clinicians

**DOI:** 10.1371/journal.pone.0128228

**Published:** 2015-07-07

**Authors:** Sabine N. van der Veer, Maria C. Haller, Carina A. C. M. Pittens, Jacqueline Broerse, Clare Castledine, Maurizio Gallieni, Nicholas Inston, Anna Marti Monros, Niels Peek, Wim van Biesen

**Affiliations:** 1 European Renal Best Practice (ERBP) Methods Support Team, University hospital Ghent, Ghent, Belgium; 2 Health e-Research Centre, Institute of Population Health, University of Manchester, Manchester, United Kingdom; 3 Center for Medical Statistics, Informatics and Intelligent Systems (CeMSIIS), Section for Clinical Biometrics, Medical University Vienna, Vienna, Austria; 4 Department for Internal Medicine III, Nephrology and Hypertension Diseases, Transplantation Medicine and Rheumatology, Krankenhaus Elisabethinen, Linz, Austria; 5 Athena Institute for Research on Innovation and Communication in Health and Life Sciences, VU university, Amsterdam, the Netherlands; 6 Sussex Kidney Unit, Brighton & Sussex University Hospital, Brighton, United Kingdom; 7 Vascular Access Society (VAS), Maastricht, the Netherlands; 8 Nephrology and Dialysis Unit, Ospedale San Carlo Borromeo, Milano, Italy; 9 Vascular Access Society of Britain and Ireland (VASBI), Glasgow, United Kingdom; 10 Department of Renal Transplantation and Renal Surgery, Queen Elizabeth Hospital Birmingham, Birmingham, United Kingdom; 11 Department of Nephrology, CHGU Valencia, Valencia, Spain; 12 Renal division, University Hospital Ghent, Ghent, Belgium; University of Milan, ITALY

## Abstract

**Background:**

Many decisions around vascular access for haemodialysis warrant a collaborative treatment decision-making process, involving both clinician and patient. Yet, patients’ experiences in this regard have been suboptimal. Although clinical practice guidelines could facilitate collaborative decision making, they often focus on the clinicians’ side of the process, while failing to address the patients’ perspective. The objective of this study was to explore and compare kidney patients’ and clinicians’ views on what vascular access-related decisions deserved priority for developing guidelines that will contribute to optimizing collaborative decision making.

**Methods:**

In the context of updating their vascular access guideline, European Renal Best Practice surveyed an international panel of 85 kidney patients, 687 nephrologists, 194 nurses, and 140 surgeons/radiologists. In an electronic questionnaire, respondents rated 42 vascular access-related topics on a 5-point Likert scale. Based on mean standardized ratings, we compared priority ratings between patients and each clinician group.

**Results:**

Selection of access type and site, as well as prevention of access infections received top priority across all respondent groups. Patients generally assigned higher priority to decisions regarding managing adverse effects of arteriovenous access and patient involvement in care, while clinicians more often prioritized decisions around sustaining patients’ access options, technical aspects of access creation, and optimizing fistula maturation and patency.

**Conclusion:**

Apart from identifying the most pressing knowledge gaps, our study provides pointers for developing guidelines that may improve healthcare professionals’ understanding of when to involve patients along the vascular access pathway.

## Introduction

Policy makers encourage collaborative decision making in health care [1–3] aimed at reaching a mutual agreement between patient and clinician on which treatment to choose, taking into account the best available evidence, as well as patients’ values and preferences [4].

Many decisions around vascular access for haemodialysis warrant a collaborative decision-making process. Firstly because numerous aspects of access creation and subsequent maintenance (e.g., selection of access type, infection prevention) may profoundly influence patients’ lifestyle and psychosocial well-being [5], or could benefit from patients’ adherence or self-management skills[6]. Furthermore, the underlying evidence base for several vascular access-related choices is weak or flawed. For example, when choosing how to preoperatively assess vessels for fistula suitability, it is uncertain whether the potential benefits of more invasive tests outweigh potential harm compared to a clinical examination [7]; and a systematic review found a high risk of selection bias in studies exploring the association between access type and outcomes [8].

Despite the apparent need for making decisions on vascular access collaboratively, previous research suggested that patients’ experiences in this regard were suboptimal [5,9]. This potentially leads to patients feeling emotionally unprepared for vascular access [5], perceiving a lack of choice regarding renal replacement therapy [9], or being reluctant to consider a fistula after starting haemodialysis on a catheter [10,11]. The suboptimal decision-making process may partly stem from the lack of support to make collaborative and informed choices around vascular access. Clinical practice guidelines have been suggested as a starting point to provide such support [12–14]. However, they often strongly focus on the clinicians’ side of decision making by only synthesizing the best available evidence, while failing to incorporate the influence of patients’ preference and their clinical and socio-personal context [15].

European Renal Best Practice (ERBP) produces guidance for health professionals who provide care to kidney patients in Europe and neighboring regions (www.european-renal-best-practice.org). Within the context of updating its 2007 vascular access guideline [16], ERBP surveyed an international panel of kidney patients and clinicians about their perspectives on what decisions related to vascular access care deserved priority for coverage by the updated guideline. Exploring and comparing both perspectives will provide pointers for developing guidance that contributes to optimizing collaborative vascular access decision making.

## Methods

**Fig 1:**
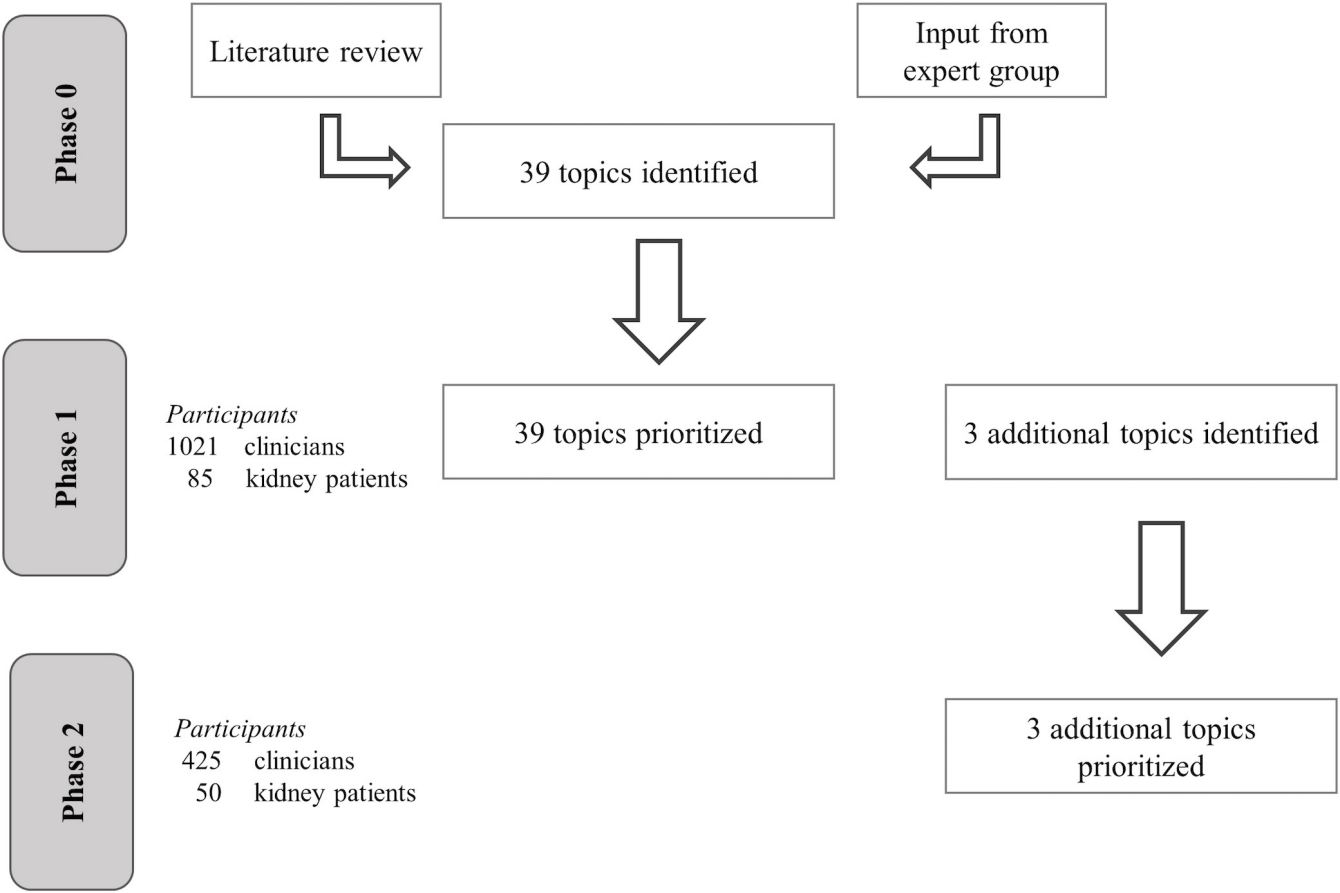
Flow chart of process of identifying and prioritizing treatment decisions (i.e., topics) around permanent vascular access

The process of identifying and prioritizing all relevant treatment decisions along the vascular access care pathway (vascular access ‘topics’) comprised three phases (Fig 1). In phase 0, we created a preliminary list of topics based on a literature review, and input from a multidisciplinary expert group. This group consisted of two kidney patients, two nephrologists, a renal nurse, two surgeons, and a radiologist. In phase 1, an international panel of clinicians and kidney patients rated the priority of these topics, as well as suggesting additional topics to complement the preliminary list. The additional topics were prioritized in phase 2.

### Developing the questionnaire

We first explored which clinical topics related to permanent vascular access care were addressed in randomized studies, systematic reviews, meta-analyses by searching the Cochrane database, combining MeSH terms and keywords related to vascular access, kidney disease, and dialysis (S1 File). Based on title and abstract, two reviewers (SV and MH) extracted the primary topic addressed in each study. They also reviewed available English language guidelines [16–19] for additional topics. Based on the exploratory review, we drafted a topic list, which the expert group reviewed and complemented; this resulted in a preliminary list of 39 topics, which respondents prioritized in the phase 1 questionnaire; they could also suggest additional topics. This generated three new topics (prioritized in phase 2), resulting in a final list of 42 topics; ten referred to general aspects of access care, 25 specifically to fistulas/grafts, and seven to permanent catheters (see S2 File). While clinicians prioritized all, patients rated only the general topics, and those referring to the access type with which they had most experience.

All participants rated the priority of topics on an unbalanced 5-point Likert scale [20], with a rating of 1 indicating ‘not important’ and the other four indicating some degree of importance (ranging from 2 ‘a little important’ to 5 ‘very important’). Patients could indicate that they were unable to rate a topic's priority, e.g., because they needed more information.

Two sets of cues [21] aimed to align the concept of priority among respondents. Clinicians’ cues focused on prioritizing topics reflecting decisions with considerable impact on patient outcomes, for which there was insufficient or conflicting evidence on what to do, or that were prevalent in clinical practice [22]. Cues for patients tried to capture topics for which respondents strongly preferred some treatment options to others while assuming medical equipoise.

All questionnaires were electronic and developed in SurveyMonkey. Questionnaires for clinicians were in English, and pilot-tested by our expert group. Patients received questionnaires professionally translated from English into their native language, and reviewed for clarity by two native speakers (one kidney patient, one clinician).

The complete questionnaires are provided as (S2 File).

### Recruitment of participants

#### Kidney patients

We recruited patients from Austria, Dutch-speaking Belgium, Spain, the Netherlands, and the United Kingdom via national patient organizations, or–in the absence of such an organization in Belgium—via nephrologists in five dialysis centres. Patients were eligible to participate if they (1) had been on haemodialysis for at least 91 consecutive days, and (2) had an e-mail address, which they accessed regularly. Finally, 101 patients consented and received an invitation for phase 1. For phase 2, we invited all patients who completed phase 1.

#### Clinicians

For phase 1 we approached various clinician groups involved in vascular access care via the European Renal Association–European Dialysis and Transplant Association, European Society of Vascular Surgery, Vascular Access Society (VAS), VAS of Britain and Ireland, and national professional networks for renal nurses in Dutch-speaking Belgium, Italy, Spain, and the Netherlands. Dissemination strategies varied between societies, including undirected e-mail blasts to all members, direct mailings, and a link in electronic newsletters or on societies’ website. For phase 2, we invited 764 clinicians from phase 1 who indicated their interest in further participation.

### Data collection and analysis

We collected data in SurveyMonkey (www.surveymonkey.com) between February and October 2014. For both surveys, participants received up to two reminders after three and six weeks.

We summarized data using descriptive statistics as appropriate. In all analyses, we distinguished patients, nephrologists, nurses, and surgeons/radiologists as separate respondent groups.

We calculated standardized ratings (rating_std_) for each individual before calculating mean ratings within each respondent group to account for the phenomenon that different people tend to use different ranges of a rating scale [20]. Per topic, we subtracted a respondent's mean rating across all topics from the original rating, and divided the result by the respondent's standard deviation [23]. Using each respondent group’s mean rating_std_, we then compared priorities between patients and each clinician group by performing two-sample t-tests per topic. To correct for multiple testing, we considered a p-value < 0.01 to indicate a statistically significant difference in priority rating.

For ease of interpretation we back-transformed the rating_std_ to the original scale for reporting priority ratings in the text and main tables (referred to as adjusted ratings, rating_adj_), as follows. We calculated the overall mean and standard deviation, using the original ratings across all topics and respondents. Subsequently, we multiplied each respondent's standardized ratings by the overall standard deviation, and added the overall mean.

We performed all analyses using IBM SPSS Statistics for Windows version 20.0.0.1.

### Ethical approval

The Research Ethics Committee of Ghent University Hospital (Ghent, Belgium) approved the study. The committees of the Academic Medical Center (Amsterdam, The Netherlands), Barts Health NHS Trust (London, UK), Consorci General University Hospital (Valencia, Spain), and Medical University of Vienna (Vienna, Austria) informed us that they did not deem formal approval necessary.

## Results

### Participants

Table 1 displays all participants' characteristics.

**Table 1:** Participants’ characteristics.^a)^

Characteristics	Number (%)^b)^
*Kidney patients (n = 85)*	
Country of residence	
Austria	13 (15)
Belgium	20 (24)
Spain	12 (14)
The Netherlands	25 (29)
United Kingdom	15 (18)
Age (years)	
< 35	10 (12)
35 to 50	23 (27)
51 to 65	35 (41)
> 65	17 (20)
Male	29 (34)
Currently on haemodialysis	74 (87)
Total time on haemodialysis (years)	
< 1	9 (11)
1 or 2	22 (26)
3 to 5	17 (20)
> 5	37 (44)
Pre-dominantly dialyzed via. …	
… an arteriovenous fistula or graft	76 (89)
…a tunneled catheter	9 (11)
*Clinicians (n = 1021)*	
Country of practice	
Spain	116 (11)
Germany	95 (9)
Italy	88 (9)
United Kingdom	77 (8)
Belgium	65 (6)
Other	580 (57)
Age (years)	
< 35	92 (9)
35 to 50	450 (44)
51 to 65	431 (42)
> 65	48 (5)
Male	654 (64)
Practising as a	
Nephrologist	687 (67)
Nurse	194 (19)
Surgeon	126 (12)
Radiologist	14 (1)
Clinical experience (years)	
< 5	79 (8)
5 to 10	153 (15)
11 to 20	311 (31)
> 20	478 (47)
Time spent in direct patient care (%)	
< 25	46 (4)
25 to 50	130 (13)
51 to 75	334 (33)
> 75	511 (50)

^a)^ Participants in phase 1

^b)^ Percentages may not add up to 100% due to rounding off

Of the 101 consenting kidney patients who received an invitation for phase 1, 85 responded (response rate [RR], 84%). The majority were 51 to 65 years of age, and female, had been on haemodialysis for more than five years, and were receiving haemodialysis at the time of the study; 76 (89%) had most experience with dialysis via a fistula or graft. The characteristics of the 50 patients who completed the phase 2 survey (RR, 59%) were similar to those participating in phase 1.

In total, 1021 clinicians from 92 countries completed the questionnaire in phase 1; our method of distribution did not allow us to ascertain a response rate. The majority were aged 35 to 50 years, male, were practicing as a nephrologist, had over twenty years of clinical experience, and spent most of their time in direct patient care. Of the 764 clinicians invited for phase 2, 425 responded (RR, 56%), with similar characteristics to clinicians in phase 1.

### Topic prioritization

In total, we included 43 782 original ratings, with an overall mean of 4.20 (standard deviation [SD], 0.46). The mean rating_adj_ and priority ranking for all topics per respondent group (S3 File), as well as all individual survey responses (S4 File) are provided.

#### Kidney patients

The overall mean for patients across all original ratings was 4.18 (SD, 0.48). On 30 occasions (<1% of all ratings) sixteen different patients indicated to be unable to rate at least one topic’s priority. Table 2 presents the ten topics to which patients assigned highest priority, with 'managing catheter thrombosis' occupying the top position. Overall, 'managing needle phobia' (mean rating_adj_, 3.69; SD, 0.57), 'catheter insertion methods' (mean rating_adj_, 3.82; SD, 0.52), and 'managing pain during cannulation' (mean rating_adj_, 3.90; SD, 0.65) received the lowest priority.

**Table 2:** Comparing priority ratings between kidney patients and clinicians for the ten topics to which *patients* assigned highest priority. Abbreviations: N, number of respondents who rated the importance of a topic; SD, standard deviation.

	KIDNEY PATIENTS			CLINICIANS								
				*Nephrologists (N = 687)*			*Nurses (N = 194)*			*Surgeons & radiologists (N = 140)*		
Topic	Rank ^a)^	N	Mean adjusted (SD) rating	Rank ^a)^	Mean adjusted (SD) rating	P-value ^b)^	Rank ^a)^	Mean adjusted (SD) rating	P-value ^b)^	Rank ^a)^	Mean adjusted (SD) rating	P-value ^b)^
Catheter thrombosis	1	9	4.47 (0.33)	14	4.33 (0.33)	0.25	11	4.33 (0.33)	0.24	28	4.12 (0.37)	**0.011**
Selection of vascular access type ^c)^	2	85	4.39 (0.29)	2	4.49 (0.31)	**< 0.01**	9	4.34 (0.37)	0.22	1	4.57 (0.24)	**< 0.001**
Training clinicians to create/maintain access	3	85	4.37 (0.41)	13	4.34 (0.34)	0.53	14	4.32 (0.34)	0.35	8	4.41 (0.36)	0.41
Catheter infection	4	9	4.36 (0.19)	1	4.53 (0.27)	0.03	1	4.49 (0.27)	0.08	18	4.30 (0.37)	0.40
Fistula/graft infection	6	76	4.35 (0.36)	4	4.40 (0.33)	0.26	3	4.41 (0.29)	0.21	16	4.31 (0.32)	0.36
Perioperative fistula/graft infection	5	76	4.34 (0.34)	22	4.27 (0.41)	0.08	4	4.40 (0.33)	0.21	23	4.20 (0.46)	**< 0.01**
Preoperative assessment of vessels	7	85	4.33 (0.40)	18	4.30 (0.38)	0.42	24	4.20 (0.41)	**0.012**	5	4.49 (0.34)	**< 0.01**
Fistula/graft-related heart disease	8	71	4.33 (0.40)	29	4.11 (0.41)	**< 0.001**	30	4.15 (0.37)	**< 0.01**	35	3.99 (0.40)	**< 0.001**
Selection of vascular access site	9	85	4.32 (0.36)	5	4.40 (0.35)	0.08	15	4.32 (0.36)	0.87	4	4.50 (0.27)	**< 0.001**
Strategies to organize vascular access care ^d)^	10	85	4.31 (0.37)	21	4.28 (0.37)	0.45	16	4.31 (0.35)	0.87	6	4.42 (0.34)	0.04

^a)^ Ranking based on mean (standard deviation) standardized ratings

^b)^ Based on two sample t-test of mean standardized ratings between patients and clinician group. P-values of <0.01 indicate a disagreement on priorities between patients and clinicians, with values between 0.010 and 0.014 being considered borderline significant.

^c)^ Topic includes subtopics such as choosing between fistula, graft and tunneled catheter; clinical and social (contra-)indications for specific access types; last resort access types

^d)^ Topic includes subtopics such as who should create fistulas; specialized vascular access centres; multidisciplinary vascular access teams; vascular access coordinators; formalized care pathways

#### Clinicians

For nephrologists, nurses, and surgeons/radiologists, the overall mean original ratings were 4.18 (SD, 0.44), 4.42 (SD, 0.38), and 3.99 (SD, 0.53), respectively. The following four topics appeared in the top 10 priority ranking of all groups: ‘selection of vascular access type’; ‘preservation of veins’; ‘management of fistula/graft stenosis’; and ‘management of central vein obstruction’. Nephrologists and nurses assigned highest priority to ‘preventing catheter infections’ (mean (SD) rating_adj_ of 4.53 (0.27) and 4.49 (0.27), respectively). Surgeons/radiologists ranked this topic 18^th^ (mean rating_adj_, 4.30; SD, 0.37), and gave top priority to ‘selection of vascular access type’ (mean rating_adj_, 4.57; SD, 0.24). ‘Management of perigraft seromas’ was at the bottom of the overall ranking for all clinician groups, with a mean (SD) rating_adj_ of 3.54 (0.61), 3.53 (0.75) and 3.58 (0.55) for nephrologists, nurses, and surgeons/radiologists, respectively.

### Differences in priority ratings between patients and clinicians

Patients’ priorities differed significantly for thirteen topics when compared to nephrologists, thirteen when compared to surgeons/radiologists, and for seven topics when compared to nurses (supplement material). In general, patients assigned higher priority than clinicians to topics regarding managing adverse effects of arteriovenous access and patient involvement in care (compared to nephrologists), while giving lower priority to decisions around preparing for access creation, the surgical procedure, and preventing poor fistula maturation. For 23 of 42 topics, priority ratings did not differ significantly between patients and any clinician group; this included five of the ten top priority topics for patients (Table 2).

#### Differences in ratings between groups for patients’ high priority topics

Table 2 shows that we found a statistically (borderline) significant difference for ten of 30 comparisons concerning patients’ top priority topics; six stemmed from patients rating topics differently from surgeons/radiologists. Patients assigned higher priority than clinicians to ‘managing fistula/graft-related heart disease’ (compared to any clinician group); ‘catheter thrombosis’ and ‘preventing perioperative fistula/graft infections’ (compared to surgeons/radiologists); and ‘preoperative assessment of vessels’ (compared to nurses). Patients assigned lower priority to ‘selection of vascular access type’ (compared to nephrologists and surgeons/radiologists), and ‘preoperative assessment of vessels’ and ‘selection of vascular access site’ (compared to surgeons/radiologists).

#### Differences in ratings between groups for clinicians’ high priority topics

Table 3 displays comparisons for the eight topics that were absent in the patient top 10, but appeared in the top 10 priority ranking of one or more clinician group. Clinicians assigned a higher priority rating than patients to ‘preservation of veins’ and ‘management of fistula/graft stenosis’ (all groups); ‘timing of vascular access creation’ and ‘surgical techniques for fistula/graft creation’ (nephrologists and surgeons/radiologists).

**Table 3:** Comparing priority ratings between kidney patients and clinicians for topics to which *clinicians* assigned high priority, but that were absent in patients’ top 10. Abbreviations: N, number of respondents who rated the importance of a topic; SD, standard deviation

	KIDNEY PATIENTS			CLINICIANS								
				*Nephrologists (N = 687)*			*Nurses (N = 194)*			*Surgeons & radiologists (N = 140)*		
Topic	N	Rank ^a)^	Mean adjusted (SD) rating	Rank ^a)^	Mean adjusted (SD) rating	P-value ^b)^	Rank ^a)^	Mean adjusted (SD) rating	P-value ^b)^	Rank ^a)^	Mean adjusted (SD) rating	P-value ^b)^
Preservation of veins	84	18	4.24 (0.51)	3	4.45 (0.36)	**< 0.01**	2	4.43 (0.33)	**< 0.01**	2	4.56 (0.34)	**< 0.001**
Central vein obstruction	9	24	4.22 (0.26)	6	4.40 (0.31)	0.07	7	4.36 (0.29)	0.13	7	4.41 (0.32)	0.06
Fistula/graft thrombosis	76	11	4.31 (0.34)	10	4.36 (0.34)	0.18	5	4.40 (0.27)	0.05	11	4.37 (0.35)	0.19
Fistula/graft stenosis	76	20	4.24 (0.35)	9	4.37 (0.34)	**< 0.01**	8	4.36 (0.29)	**< 0.01**	10	4.38 (0.34)	**< 0.01**
Surveillance of fistula/graft (dys)function	76	14	4.26 (0.30)	12	4.34 (0.35)	0.06	10	4.33 (0.34)	0.12	12	4.33 (0.35)	0.14
Surgical techniques for fistula/graft creation	75	33	4.10 (0.43)	8	4.39 (0.35)	**< 0.001**	21	4.24 (0.43)	0.02	3	4.54 (0.28)	**< 0.001**
Timing of vascular access creation	84	26	4.21 (0.39)	7	4.39 (0.35)	**< 0.001**	22	4.24 (0.36)	0.50	9	4.38 (0.33)	**0.013**
Perioperative fistula/graft thrombosis	75	12	4.30 (0.37)	20	4.29 (0.38)	0.71	6	4.37 (0.34)	0.18	21	4.26 (0.35)	0.41

^a)^ Ranking based on mean (standard deviation) standardized ratings

^b)^ Based on two sample t-test of mean standardized ratings between patients and clinician group. P-values of <0.01 indicate a disagreement on priorities between patients and clinicians, with values between 0.010 and 0.014 being considered borderline significant.

For the remaining four topics in Table 3, we found no evidence for differing priority ratings.

## Discussion

### Summary of findings

In this study, we explored and compared kidney patients' and clinicians' perspective on what decisions related to vascular access care deserved priority for coverage by an international guideline. Selection of access type and site, as well as preventing access infections received high priority across respondent groups. Patients generally assigned higher priority to decisions regarding managing adverse effects of arteriovenous access and patient involvement in care, while clinicians more often prioritized decisions around sustaining patients’ access options, technical aspects of access creation, and optimizing fistula maturation and patency.

### Top priorities for optimizing vascular access decision making

When taking into account both the patient and clinician perspective, the following four vascular access-related decisions emerged as most important: access type selection, access site selection, preventing catheter infection, and preventing fistula/graft infection. These topics should get top priority for the updated ERBP guideline. As part of an international harmonization initiative among seven renal guideline bodies [24], ERBP intends to update the systematic reviews underlying the Caring for Australasians with Renal Impairment (CARI) vascular access guidelines [25–27]. Apart from informing the clinical side of the decision-making process, the updated evidence synthesis will allow identification of persistent gaps in knowledge. Current research collaboratives focus on improving fistula maturation and patency [28,29], and our study confirms the relevance of these topics from the clinician perspective. However, based on our findings, health policy makers should additionally consider encouraging initiatives aimed at improving patient outcomes for any access type (not just fistulas), and development of more advanced (statistical) research methods to address the risk of selection bias in non-randomized studies comparing outcomes between access types [8].

In keeping with previous studies, our results highlight a strong patient preference regarding access type and site, and the management of access infections [5,10,11,30,31]. To communicate this, Van der Weijden *et al*. suggested that guidelines should mark these and other patient-priority topics as decisions requiring elicitation of patient preferences [14]. This preference elicitation could be accommodated by extending guideline recommendations with information on the patient perspective, or by developing a guideline-based patient decision aid covering these topics [13,14,32]. Readily available qualitative research, such as the recent qualitative evidence synthesis by Casey *et al*. [5], may serve as a point of departure.

### Differing priorities between patients and clinicians

Clinicians assigned less priority than patients to decisions around managing adverse effects of arteriovenous access (e.g., fistula/graft-related heart disease) and catheter thrombosis (only surgeons/radiologists). These lower ratings may reflect the relatively low prevalence of such complications in clinicians’ daily practice. Nephrologists also gave lower ratings to topics regarding patient involvement in care, potentially indicating unawareness of patient involvement’s potential to improve outcomes [33]. Others might not recognize the uncertainty around optimal kidney patient involvement in vascular access decisions, or the persistent room for improvement [5,9]. Future studies investigating nephrologists’ knowledge on and attitude towards patient involvement will help to design interventions that foster collaborative vascular access decision making [34].

In contrast, preservation of veins received lower priority from patients than from clinicians. This implies that patients do not strongly prefer certain vein preservation strategies to others, and that they would accept the strategy recommended by their doctor. At the same time, our results suggest that clinicians would like a more robust evidence base for recommending how to preserve veins for fistula creation, making this an important topic for guideline development. Patients also gave relatively low priority to topics related to the surgical procedure of arteriovenous access creation. Since we asked patient respondents to assume medical equipoise between treatment options, one explanation for the lack of preference might be that they do not expect choices around access surgery, such as selecting a particular vein for fistula creation or choosing between a synthetic and an autologous graft, to directly affect their lives.

### Strengths and limitations

To our knowledge, this is the first study to explore both clinician and kidney patient priorities on all decisions along the vascular access care pathway. Previous studies exploring patients’ perspectives on vascular access [5,10,11,30,31] focused mainly on the choice of access type, and/or did not allow prioritization of patient preference-sensitive decisions; none of them permitted a direct comparison with clinicians’ priorities. Our international study sample together with our quantitative approach and analysis strategy, enabled us to draw a detailed and robust picture of priorities among the main stakeholders in vascular access care. This picture complements the results from a recent study in which Canadian clinicians, patients and caregivers identified vascular access as one of the research priorities within the management of kidney failure [35]. Furthermore, we suggest that collaborating with kidney patients in developing and executing the survey contributed to successful patient involvement in the early stages of guideline development, which is advocated by broadly accepted guideline development standards [36–38].

Our study population likely represented a selected sample of clinicians and kidney patients. This limits the generalizability of our findings, particularly those regarding patient priorities. Patient respondents had to be able and willing to participate, understand the information in the survey, use a computer and email account, and be active in patient organizations. Since patient characteristics, such as higher educational attainment and better self-reported health, potentially increase patients’ desire to be involved in treatment selection [39], this might have resulted in our overestimating the strength of patients’ preferences for some decisions. Selection bias may also explain the low number of patients with a permanent catheter in our sample. This decreased the reliability of patient ratings for the seven catheter-related topics, and thus the probability of finding differences with clinicians' ratings. Still, the patient ranks were largely comparable to those for similar fistula-related topics. Lastly, kidney patients may be more familiar with common treatments, for example those for managing prevalent complications. Having personal experience with particular treatments potentially affects whether one prefers those treatments to others, with preference strength either increasing or decreasing, depending on the experience itself. Since patients did not report on previous experiences with vascular access care, we were unable to assess how this influenced our findings.

In conclusion, this study provides pointers for optimizing a collaborative decision-making process around vascular access for haemodialysis. In addition to identifying the most pressing knowledge gaps in access care, the findings contribute to developing guidelines that could ultimately improve healthcare professionals’ understanding of when and how to involve kidney patients in decisions on the vascular access care pathway.

## Supporting Information

S1 File Terms used for searching the Cochrane database in phase 0. (PDF)

S2 File Complete questionnaires. (PDF)

S3 File Mean adjusted ratings and ranks of all 42 vascular access topics included in the survey. Results are reported per respondent group.
(PDF)

S4 File Individual survey responses. (SAV)

## References

[1] Patient Protection and Affordable Care Act. 111th United States Congress; 2010 pp. Public law no. 111–148,§3506, 124 Stat. p119–1025.

[2] Health and Social Care Act [Internet]. Parliament of the United Kingdom; 2012 p. Chapter 7. Available: http://www.legislation.gov.uk/ukpga/2012/7/contents/enacted

[3] Institute of Medicine. Crossing the quality chasm Washington, D.C.: The National Academies Press; 2001.

[4] Charles C, Gafni A, Whelan T. Decision-making in the physician-patient encounter: revisiting the shared treatment decision-making model. Soc Sci Med. 1999;49: 651–61. 10452420 10.1016/s0277-9536(99)00145-8

[5] Casey JR, Hanson CS, Winkelmayer WC, Craig JC, Palmer S, Strippoli GFM, et al. Patients’ perspectives on hemodialysis vascular access: a systematic review of qualitative studies. Am J Kidney Dis. Elsevier Inc; 2014; 10.1053/j.ajkd.2014.06.024 25115617

[6] Sousa CN, Apóstolo JL, Figueiredo MH, Martins MM, Dias VF. Interventions to promote self-care of people with arteriovenous fistula. J Clin Nurs. 2014;23: 1796–802. 10.1111/jocn.12207 23773233

[7] Wong CS, McNicholas N, Healy D, Clarke-Moloney M, Coffey JC, Grace P a, et al A systematic review of preoperative duplex ultrasonography and arteriovenous fistula formation. J Vasc Surg. Society for Vascular Surgery; 2013;57: 1129–33. 10.1016/j.jvs.2012.11.094 23535043

[8] Ravani P, Palmer SC, Oliver MJ, Quinn RR, MacRae JM, Tai DJ, et al Associations between hemodialysis access type and clinical outcomes: a systematic review. J Am Soc Nephrol. 2013;24: 465–73. 10.1681/ASN.2012070643 23431075 PMC3582202

[9] Morton RL, Tong A, Howard K, Snelling P, Webster AC. The views of patients and carers in treatment decision making for chronic kidney disease: systematic review and thematic synthesis of qualitative studies. BMJ. 2010;340: c112 10.1136/bmj.c112 20085970 PMC2808468

[10] Fissell RB, Fuller DS, Morgenstern H, Gillespie BW, Mendelssohn DC, Rayner HC, et al Hemodialysis patient preference for type of vascular access: variation and predictors across countries in the DOPPS. J Vasc Access. 2013;14: 264–72. 10.5301/jva.5000140 23599135

[11] Xi W, Harwood L, Diamant MJ, Brown JB, Gallo K, Sontrop JM, et al Patient attitudes towards the arteriovenous fistula: a qualitative study on vascular access decision making. Nephrol Dial Transplant. 2011;26: 3302–8. 10.1093/ndt/gfr055 21406543

[12] Van Biesen W, van der Veer SN, Jager KJ, Fouque D, Wanner C, Vanholder R. What guidelines should or should not be: implications for guideline production. Nephrol Dial Transplant. 2013;28: 1980–4. 10.1093/ndt/gft291 23990354

[13] Raats CJI, van Veenendaal H, Versluijs MM, Burgers JS. A generic tool for development of decision aids based on clinical practice guidelines. Patient Educ Couns. 2008;73: 413–7. 10.1016/j.pec.2008.07.038 18768285

[14] Van der Weijden T, Pieterse AH, Koelewijn-van Loon MS, Knaapen L, Légaré F, Boivin A, et al How can clinical practice guidelines be adapted to facilitate shared decision making? A qualitative key-informant study. BMJ Qual Saf. 2013;22: 855–63. 10.1136/bmjqs-2012-001502 23748154

[15] Wyatt KD, Stuart LM, Brito JP, Carranza Leon B, Domecq JP, Prutsky GJ, et al Out of context: clinical practice guidelines and patients with multiple chronic conditions: a systematic review. Med Care. 2014;52 Suppl 3: S92–S100. 10.1097/MLR.0b013e3182a51b3d 23969592

[16] Tordoir J, Canaud B, Haage P, Konner K, Basci A, Fouque D, et al EBPG on Vascular Access. Nephrol Dial Transplant. 2007;22 Suppl 2: ii88–117. 10.1093/ndt/gfm021 17507428

[17] Polkinghorne KR, Chin GK, MacGinley RJ, Owen AR, Russell C, Talaulikar GS, et al KHA-CARI Guideline: vascular access—central venous catheters, arteriovenous fistulae and arteriovenous grafts. Nephrol. 2013;18: 701–5. 10.1111/nep.12132 23855977

[18] Fluck R, Kumwenda M. UK Renal Association clinical practice guidelines vascular access for haemodialysis [Internet]. 2011 p. (5th edition). Available: http://www.renal.org/guidelines/modules/vascular-access-for-haemodialysis 10.1159/000328071 21555898

[19] National Kidney Foundation Kidney Disease Outcomes Quality Initiative. 2006 Updates Clinical Practice Guidelines Vascular Access [Internet]. 2006 pp. 227–409. Available: http://www2.kidney.org/professionals/KDOQI/guideline_upHD_PD_VA

[20] Streiner DL, Norman GR. Health Measurement Scales A practical guide to their development and use. 3rd ed Oxford (United Kingdom): Oxford University Press; 2003 pp. p93–94.

[21] Murphy M, Black N, Lamping D, McKee C, Sanderson C, Askham J, et al Consensus development methods, and their use in clinical guideline development. Health Technol Assess (Rockv). 1998;2. 9561895

[22] Eccles MP, Grimshaw JM, Shekelle P, Schünemann HJ, Woolf S. Developing clinical practice guidelines: target audiences, identifying topics for guidelines, guideline group composition and functioning and conflicts of interest. Implement Sci. 2012;7: 60 10.1186/1748-5908-7-60 22762776 PMC3523009

[23] Altman DG. Practical statistics for medical research 1st ed London (UK): Chapman & Hall; 1991 pp. 54–60.

[24] Haller MC, van der Veer SN, Nagler E V, Tomson C, Lewington A, Hemmelgarn BR, et al. A survey on the methodological processes and policies of renal guideline groups as a first step to harmonize renal guidelines. Nephrol Dial Transplant. 2014; [ePub ahead of print]. 10.1093/ndt/gfu288 25204317

[25] Lopez-Vargas P, Polkinghorne K. KHA-CARI guideline for Selection of type of access [Internet]. 2012. Available: http://www.cari.org.au/Dialysis/dialysis_guidelines.html

[26] Lopez-Vargas P, Polkinghorne K. KHA-CARI guideline for Nursing care of central venous catheters [Internet]. 2012. Available: http://www.cari.org.au/Dialysis/dialysis_guidelines.html

[27] Chin GK. KHA-CARI guideline for Prevention of tunnelled dialysis catheter infection [Internet]. 2012. Available: http://www.cari.org.au/Dialysis/dialysis_guidelines.html

[28] Dember LM, Imrey PB, Beck GJ, Cheung AK, Himmelfarb J, Huber TS, et al Objectives and design of the hemodialysis fistula maturation study. Am J Kidney Dis. Elsevier Inc; 2014;63: 104–12. 10.1053/j.ajkd.2013.06.024 23992885 PMC4134933

[29] ReDVA research collaboration. Development of hemodynamic solutions in Renal Dialysis Venous Access failure [Internet]. 2013. Available: http://www.redva.eu/

[30] Moura A, Madureira J, Alija P, Fernandes J, Oliviera J, Lopez M, et al Type of vascular access and location in online hemodiafiltration and its association with patient’s perception of health-related quality of life. J Vasc Access. 2014;15: 175–82. 10.5301/jva.5000182 24170586

[31] Quinn R, Lamping D, Lok CE, Meyer R, Hiller J, Lee J, et al The vascular access questionnaire: assessing patient-reported views of vascular access. J Vasc Access. 2008;9: 122–8. 18609528

[32] Tong A, Lopez-Vargas P, Howell M, Phoon R, Johnson D, Campbell D, et al Consumer involvement in topic and outcome selection in the development of clinical practice guidelines. Heal Expect. 2012;15: 410–23. 10.1111/j.1369-7625.2011.00676.x PMC5060636 23134217

[33] Ahmad N, Ellins J, Krelle H, Lawrie M. Person-centred care : from ideas to action Bringing together the evidence on shared decision making and self-management support. The Health Foundation, London (UK); 2014.

[34] Légaré F, Ratté S, Gravel K, Graham ID. Barriers and facilitators to implementing shared decision-making in clinical practice: update of a systematic review of health professionals’ perceptions. Patient Educ Couns. 2008;73: 526–35. 10.1016/j.pec.2008.07.018 18752915

[35] Manns B, Hemmelgarn B, Lillie E, Dip SCPG, Cyr A, Gladish M, et al Setting research priorities for patients on or nearing dialysis. Clin J Am Soc Nephrol. 2014;9: 1813–21. 10.2215/CJN.01610214 24832095 PMC4186509

[36] Brouwers MC, Kho ME, Browman GP, Burgers JS, Cluzeau F, Feder G, et al AGREE II: advancing guideline development, reporting and evaluation in health care. J Clin Epidemiol. 2010;63: 1308–11. 10.1016/j.jclinepi.2010.07.001 20656455

[37] Institute of Medicine. Clinical practice guidelines we can trust Graham R, Mancher M, Wolman DM, Greenfield S, Steinberg E, editors. Washington, D.C.: The national academies press; 2011. 24983061

[38] Guideline International Network (G-I-N). G-I-N PUBLIC Toolkit : Patient and public involvement in guidelines [Internet]. 2012. Available: http://www.g-i-n.net/working-groups/gin-public/toolkit

[39] Flynn KE, Smith M a, Vanness D. A typology of preferences for participation in healthcare decision making. Soc Sci Med. 2006;63: 1158–69. 10.1016/j.socscimed.2006.03.030 16697096 PMC1637042

