# Supplementary material for: Setting Priorities for Optimizing Vascular Access Decision Making – An International Survey of Patients and Clinicians
Source: PLoS One. 2015 Jul 7;10(7):e0128228. doi: 10.1371/journal.pone.0128228 (PMC4494812; doi:10.1371/journal.pone.0128228)
Supplement: S1 File — (PDF) [file pone.0128228.s001.pdf]

S1 File: Terms used for searching the Cochrane database in phase 0

1. arteriovenous next shunt$.ti
2. arteriovenous shunt$.ti
3. arteriovenous next shunt$.ab
4. arteriovenous shunt$.ab
5. arteriovenous next shunt$.kw
6. arteriovenous shunt$.kw
7. exp Arteriovenous Shunt, Surgical/
8. arteriovenous next fistula.ti
9. arteriovenous fistula.ti
10. arteriovenous fistula.ab
11. arteriovenous fistula.kw
12. exp Arteriovenous Fistula/
13. blood next vessel next prothesis.ti
14. blood near vessel near prothesis.ti
15. (blood adj3 vessel adj3 prothesis).ti
16. blood vessel prothesis.ti
17. blood vessel prothesis.ab
18. blood vessel prothesis.kw
19. Exp Blood Vessel Prothesis/
20. (fistula$ or AVF$ or shunt or shunts).ti
21. (fistula$ or AVF$ or shunt or shunts).ab
22. (fistula$ or AVF$ or shunt or shunts).kw
23. (vascular next access or venous next access).ti
24. (vascular next access or venous next access).ab
25. (vascular next access or venous next access).kw
26. vascular access.ti
27. vascular access.ab
28. vascular access.kw
29. venous access.ti
30. venous access.ab
31. venous access.kw
32. venous access.tw
33. exp Catheterization, Central Venous/
34. {OR 1-33}
35. exp Renal Replacement Therapy/
36. renal replacement therapy.tw.
37. dialysis.tw.
38. dialysis.ti.
39. dialysis.ab.
40. dialysis.kw.
41. exp Renal Dialysis/
42. (predialysis or pre-dialysis).ti.
43. (predialysis or pre-dialysis).ab.
44. (predialysis or pre-dialysis).kw.
45. (haemodialysis or hemodialysis).ti.
46. (haemodialysis or hemodialysis).ab.
47. (haemodialysis or hemodialysis).kw.
48. (kidney adj5 disease).ti.

49. (kidney adj5 disease).ab.
50. (kidney adj5 disease).kw.
51. kidney next disease.ti.
52. (kidney adj5 failure).ti.
53. (kidney adj5 failure).ab.
54. (kidney adj5 failure).kw.
55. (renal adj5 insufficiency).ti.
56. (renal adj5 insufficiency).ab.
57. (renal adj5 insufficiency).kw.
58. (CRF or CRD or CKF or CKD or ESRD or ESRF or ESKF or ESKD).ab.
59. (CRF or CRD or CKF or CKD or ESRD or ESRF or ESKF or ESKD).ti.
60. (CRF or CRD or CKF or CKD or ESRD or ESRF or ESKF or ESKD).kw.
61. {OR 35 - 60}
62. 34 and 61
