# Supplementary material for: Setting Priorities for Optimizing Vascular Access Decision Making – An International Survey of Patients and Clinicians
Source: PLoS One. 2015 Jul 7;10(7):e0128228. doi: 10.1371/journal.pone.0128228 (PMC4494812; doi:10.1371/journal.pone.0128228)
Supplement: S2 File — (PDF) [file pone.0128228.s002.pdf]

S2 File: Complete questionnaires

CLINICIANS' QUESTIONNAIRE

Welcome to the survey on "Permanent vascular access in haemodialysis patients". It is the first step of an extensive project in which European Renal Best Practice (ERBP) will develop a new international guideline on this topic in collaboration with several other professional societies in Europe.

The survey consists of two parts:
Part 1 contains 10 questions about you and the institution where you practise.
Part 2 asks you to state which topics you as a health care professional consider high priority within your practice. Based on your input, we will define which topics are relevant for being addressed in the new guideline.

Your responses will be anonymised and remain confidential. They are automatically saved when you go to a next page in the survey. It is possible to exit the survey and return at a later time to complete or adjust your response, but only if you use the same computer and the same internet browser.

The estimated time for completing the survey is 10 to 15 minutes.

Part 1: About you

You are currently practising as a ...

- ○ ... interventional nephrologist
- ○ ... (interventional) radiologist
- ○ ... nephrologist
- ○ ... nurse (renal, vascular surgery, etc.)
- ○ ...surgeon (vascular, cardiovascular, transplant, etc.)
- ○ Other (please specify)

How many years have you been practising as a health care professional? (excluding training time)

- Less than 5
- 5 to 10
- 10 to 20
- More than 20

What percentage of your time do you spend in direct patient care?

- Less than 25%
- 25 to 50%
- 51 to 75%
- More than 75%

In what country do you practise?

...

What is your current age (in years)?

- Younger than 35
- 35 to 50
- 51 to 65
- Older than 65

What is your gender?

- Female
- Male

Is the institution of your primary affiliation...

...associated with a university?

- Yes
- No

...hospital based?

- Yes
- No

...not for profit?

- Yes
- No

...private?

○ Yes

○ No

Part 2: Prioritising potential guideline topics

The aim of this section is to determine which topics should have the highest priority for being addressed in the new European guideline on "Permanent vascular access in haemodialysis patients".

Based on a systematic literature search and expert input, we created a list of 39 potential clinical topics,
which we organised into the following 4 categories:
1. Patient preparation for access creation and vascular access type selection
2. Arteriovenous fistulae and grafts
3. Tunnelled, cuffed haemodialysis catheters
4. Organisational aspects of providing vascular access care

For each topic, we ask you to rate its priority on a scale ranging from 1 (not important) to 5 (very important). Please assign the highest priority to those topics for which having a robust evidence base is most relevant within your clinical practice. At the end of this part of the survey, you get the opportunity to suggest additional high priority topics that you did not find in any of the categories.

Assign the highest priority to topics referring to clinical situations, procedures or decisions regarding vascular access care in haemodialysis patients:

1. that are common in your daily practice or
2. that strongly affect the outcomes of your patients (e.g., quality of life, survival) or
3. for which there is uncertainty or disagreement around optimal care or
4. that are associated with high costs for your patient (side effects, adverse events) or for the healthcare system (resource use).

Definitions and abbreviations in this section

AV = Arteriovenous

Catheter = Tunnelled, cuffed haemodialysis catheter

Topic	*Not important*	*A little important*	*Moderately important*	*Important*	*Very important*
CATEGORY 1: PATIENT PREPARATION FOR ACCESS CREATION AND VASCULAR ACCESS TYPE SELECTION					
**1. Preservation of veins**					
**2. Timing of vascular access creation** (e.g., when to start talking about it with the patient, when to first assess the vessels, time of surgical referral)					
**3. Preoperative assessment of vessels** (e.g., general clinical evaluation, imaging techniques for vein mapping, cut off values for suitable vessels)					
**4. Patient involvement in preparing for access creation and type selection** (e.g., educational strategies, decision-making aids, psychosocial support)					
**5. Selection of vascular access type** (e.g., tunneled catheter versus AV fistula versus AV graft, clinical and social (contra-) indications for specific access types, last resort access types)					
**6. Selection of vascular access site** (e.g., upper versus lower limb for fistulas/grafts, internal jugular versus subclavian vein for catheters)					
CATEGORY 2: ARTERIOVENOUS (AV) FISTULAE AND GRAFTS					
Surgical procedure to create AV fistulae and grafts					
7. **Surgical techniques** (e.g., selection of fistula or graft type, microsurgery, use of vessel dilators					
**8. Selection of AV graft material** (e.g., synthetic versus autologous, hybrid grafts)					
**9. Timing of surgical procedure** (i.e., when to create the access)					
**10. Local regional versus general anesthesia**					
**11. Prevention, diagnosis and treatment of peri-operative fistula thrombosis** (i.e., during and shortly after surgery)					
**12. Prevention of peri-operative infections**					
Maturation of AV fistulae					
**13. Defining and assessing AV fistula maturation**					
**14. Prevention of poor AV fistula maturation** (e.g., indications for preemptive interventions, forearm or upper limb exercise, obliteration of venous branches)					
15. **Interventions for poor AV fistula maturation** (e.g., surgery, angiograpy)					
Cannulation of AV fistulae and grafts					
**16. Timing of first cannulation**					
**17. Selection of cannula type** (e.g., needle gauge, needle material, using smaller needles for first cannulation)					
**18. Managing pain during cannulation**					
**19. Managing needle phobia**					
**20. Cannulation procedure** (e.g., button hole versus rope ladder technique, needle orientation, use of imaging techniques, self-cannulation, needle fastening during dialysis in order to avoid dislodgment, tourniquet use, needling of deep fistulas, surveillance of puncture site)					

Topic	*Not important*	*A little important*	*Moderately important*	*Important*	*Very important*
21. **Establishing hemostasis after cannulation** (e.g., use of hemostatic plaster, use of clamps, management of malpuncture incidents)					
AV fistulae and grafts complications					
22. **Surveillance of fistula/graft (dys)function** (e.g., methods, frequency, cut-off values for blood flow/venous pressure/recirculation)					
23. **Prevention, diagnosis and treatment of stenosis** (e.g., preemptive invasive interventions, interventional radiology, timing of interventions)					
24. **Prevention, diagnosis and treatment of thrombosis** (e.g., oral anticoagulants, effect of medication (ESAs, statins) on thrombosis risk, preemptive invasive interventions, thrombectomy, timing of interventions)					
25. **Prevention and diagnosis of infection** (e.g., aseptic techniques before cannulation, prophylactic antibiotics)					
26. **Prevention, diagnosis and treatment of perigraft seromas**					
AV fistulae and grafts adverse effects					
27. **Prevention and treatment of AV access related heart disease**					
28. **Diagnosis and treatment in case of atypical arm/hand pain**					
29. **Prevention and treatment of limb ischemia** (e.g., diagnosis/treatment of steal syndrome)					
30. **Prevention and treatment of aneurysms**					
31. **Prevention and treatment of carpal tunnel syndrome**					
CATEGORY 3: TUNNELED, CUFFED HAEMODIALYSIS CATHETER					
Catheter insertion					
32. **Selection of catheter type, material, and site** (e.g., coating, antegrade/retrograde tunneling, single/double lumen)					
33. **Insertion methods** (e.g., ultrasound guided versus anatomic landmarks)					
34. **Verification of catheter tip position**					
Catheter complications and adverse effects					
35. **Surveillance of catheter (dys)function** (e.g., methods, frequency, cut-off values for blood flow, long-term surveillance)					
36. **Prevention, diagnosis and treatment of thrombosis** (e.g., anticoagulants, lock solutions, catheter exchange, timing of interventions)					
37. **Prevention and diagnosis of catheter infections** (e.g., (dis)connection techniques, exit site care, taking showers, lock solutions, (timing of) catheter exchange)					
38. **Prevention and treatment of central vein obstruction**					

Topic	*Not important*	*A little important*	*Moderately important*	*Important*	*Very important*
CATEGORY 4: ORGANIZATIONAL ASPECTS OF PROVIDING VASCULAR ACCESS					
**39. Training, certification and monitoring of skills/expertise of health care professionals in creation and maintenance** (e.g., who should be trained for what, educational strategies, minimum requirements for number of procedures per surgeon)					
**40. Strategies to organise vascular access care** (e.g., multidisciplinary teams, vascular access coordinators, who should create fistulas, care pathways, dedicated surgical resources, specialised vascular access centres)					
**41. Involvement of patient and family in access maintenance** (e.g., promoting self-management, educational programmes, decision-making aids)					
**42. Monitoring and improving the quality of vascular access care** (e.g., performance feedback, quality audits, financial incentives, meaningful performance indicators)					

Suggestions for additional topics

Do you have suggestions for additional topics that should have high priority for being addressed in the new European guideline on "Permanent vascular access in haemodialysis patients"?

O Yes

O No

{This question was only asked if respondents answered 'yes' to the previous question}

Your suggestions for additional high priority topics:

Suggestion 1: ...

Suggestion 2: ...

Suggestion 3: ...

PATIENTS' QUESTIONNAIRE

The survey consists of two parts:

Part 1 7 questions about you and your treatment

Part 2 your personal opinion on the importance of several aspects of vascular access care

We suggest you complete the survey in one sitting. Your responses will be stored automatically when going to a next page in the survey. If needed, you can exit the survey and return at a later time to complete or adjust your answers, but only if you use the same computer and the same internet browser .

PART 1 – About you and your treatment

Have you ever received haemodialysis for at least 90 days in a row?

- O Yes
- O No

Are you currently on haemodialysis?

- O Yes
- O No

In total, how many years have you been on haemodialysis?(if you have been on and off haemodialysis, please add up the time of the separate episodes)

- O Less than 1 year
- O 1 to 3 years
- O 3 to 5 years
- O More than 5 years

Taking into account all the years you have been on haemodialysis, did you predominantly dialyse at home or in a dialysis centre?

- O Home
- O In-centre

What is your current age (in years)?

- O Younger than 35
- O 35 to 50
- O 51 to 65
- O Older than 65

Are you male or female?

- O Female
- O Male

Is the dialysis centre where you currently receive treatment ...

...a private clinic?
- O Yes
- O No

...**based in a hospital?**
- O Yes
- O No

{This question was only asked if respondents answered 'yes' to the previous question}
... **Is it an academic hospital?**
- O Yes
- O No

PART 2 – Which topics do you feel are important to include in the guideline?

Instructions for completion

In this section you can give your opinion –based on your personal experience– on which topics are important to be covered by the new vascular access guideline. We ask you to rate a list of topics on a scale ranging from 1 (not important) to 5 (very important). For each topic in the list we give examples of some of the possible treatment options your doctor might suggest. It is not a problem if you lack actual experience with some of them. At the end of the section, you can suggest additional high priority topics that you did not find in the list.

How to rate the importance of a topic: an example

Selection of vascular access site (where in your body your access is placed)
Possible options are: In your left or right arm; Wrist or elbow; In your left or right leg (for fistulas/grafts); In your neck or groin; In your left or right leg (for catheters).

The topic 'Selection of vascular access site' is displayed in **bold**, followed by an explanation of the topic between brackets. In the smaller, grey text below the topic are examples of possible treatment options. If available, we added pictures or illustrations {*authors note: all images in the original questionnaire have been removed in this file due to copyright restrictions*}.

In order for you to decide on the importance of this topic, first assume that all options are equally safe and effective (even though this is not always the case). Then ask yourself the following two questions:
'How strongly do I prefer one option over the others?', and
'How much effort would it take my doctor to convince me that the other options are better?'.

If –for whatever reason– you have a strong preference for having the fistula placed in one of your legs, your kidney doctor should make a substantial effort to convince you that it is better to have a fistula in your arm. In that case, you should rate this topic as 'important' or 'very important', depending on the strength of your preference.

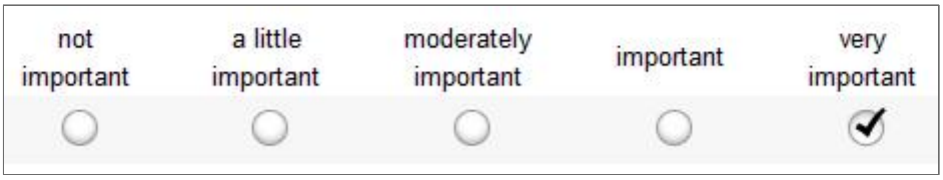

If you do not have any preference for one particular access site, your doctor does not need to convince you that one option is better than the others. You will accept to have the fistula placed in your right leg just as easily as in your left wrist or right elbow; whatever your doctor thinks it is the best place. In this case, you should rate this topic as 'not important'.

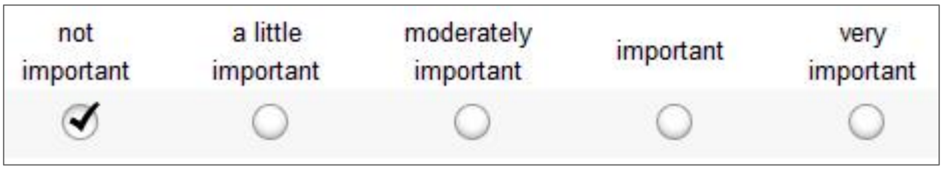

Important! If you are not sure if you have a preference or not, for example because you need more information about the possible options, choose 'not important'.

Your care when preparing you for a vascular access procedure

Once you opted for haemodialysis, there is ideally sufficient time to select the best vascular access and prepare it for the first dialysis session. The topics listed below all concern aspects of your treatment during this selection and preparation phase.

Please rate the importance of each of the following topics

For each topic, first assume that all options are equally safe and effective. Then ask yourself: *'How strongly do I prefer one option over the others?',* and *'How much effort would it take my doctor to convince me that the other options are better?'*.

1. **Preservation of veins** (protecting the blood vessels where the fistula/graft will be made)

Possible options are: (how) Doing nothing; Not allowing anyone to draw blood or give an injection in your access arm; Wearing a medical alert bracelet to inform hospital staff about your arm; (when) As soon as you are diagnosed with kidney disease; In the six months before the fistula/graft is placed;

O	O	O	O	O
Not important	A little important	Moderately important	Important	Very important

2. **Timing of vascular access creation** (timing of each step in the preparation process)

Possible options are: Taking all preparation steps as early as possible *versus* postponing them until it is clear that/when you will need haemodialysis. For example, discussing vascular access during the first consultation with your kidney doctor *versus* postponing it until your first haemodialysis session is planned.

O	O	O	O	O
Not important	A little important	Moderately important	Important	Very important

3. **Preoperative assessment of vessels** (examination of your arteries and veins to see if they are suitable for placing a fistula/graft)

Possible options are: Talking with your doctor (about your life style, medical history, previous experiences with vascular access, etc.); Physical examination of your arms by your doctor; Examination of your vessels with ultrasound; Examination of your vessels with X-ray or MRI scan after injection of contrast dye.

O	O	O	O	O
Not important	A little important	Moderately important	Important	Very important

4. **Patient involvement in preparing for access creation and type selection** (the support you get for playing an active role in selecting and preparing your vascular access)

Possible options are: Patient folders on the pros and cons of each access type; You and your doctor talking about different vascular access options using a structured list of other patients' frequently asked questions; A DVD on what you can do yourself to prepare for having a fistula/graft placed.

O	O	O	O	O
Not important	A little important	Moderately important	Important	Very important

5. **Selection of vascular access type** (choosing between a fistula/graft and a catheter)

Arteriovenous (AV) fistula or graft (also sometimes called shunt): involves the creation of a permanent connection between an artery (vessel that brings blood from the heart to the tissues) and a vein (blood vessel that returns the blood to the heart) under the skin. For fistulas this is done by directly connecting a vein to a nearby artery, while for grafts a tube is used to connect a vein with an artery. Fistulas and grafts are usually placed in the arm, but may also be placed in the leg.

Tunneled catheter (also called a line): a tube placed directly into a large vein (typically in the neck, but sometimes in the groin), which is tunneled beneath the skin for approximately 3-8 cm.

Prominent characteristics of fistulae/grafts compared to catheters: May require more than one operation to get a functioning access; Takes some time after the operation before it can be used; Situated completely underneath the skin; May create (big) bulges on your arm; Needles are inserted for each dialysis session; Once matured, it is more likely to get sufficient blood flow for dialysis; Less prone to infections; You can shower and swim; If severely damaged, it can cause a life-threatening bleed.

O	O	O	O	O
Not important	A little important	Moderately important	Important	Very important

6. **Selection of vascular access site** (where in your body your access is placed)

Possible options are: In your left or right arm; Wrist or elbow; In your left or right leg (for fistulae/grafts); In your neck or upper chest; In your left or right leg (for catheters).

O	O	O	O	O
Not important	A little important	Moderately important	Important	Very important

Your vascular access

Which type of vascular access have you had the longest?

- O Arteriovenous fistula
- O Arteriovenous graft
- O Tunneled catheter

How long did you have this vascular access?

- O Less than one year
- O 1 to 3 years
- O More than 3 years

Have you had any other type of vascular access during your time on haemodialysis than the one you already selected? (tick all that apply)

- ☐ Arteriovenous fistula
- ☐ Arteriovenous graft
- ☐ Tunneled catheter
- ☐ No

Your care around the operation date and once the access is in place – Arteriovenous (AV) fistulae and grafts

The topics in this section all concern aspects of the operation to place a fistula/graft as your vascular access, the process of getting it ready for the first haemodialysis session, and the care once you are using it for dialysis.

How many operations for creating your vascular access did you have before you had an access that was suitable for haemodialysis?

- O 1
- O 2
- O 3
- O More than 3

Do you have experience with inserting the dialysis needles yourself?

- O Yes
- O No

Please rate the importance of each of the following topics

For each topic, first assume that all options are equally safe and effective. Then ask yourself: *'How strongly do I prefer one option over the others?'*, and *'How much effort would it take my doctor to convince me that the other options are better?'*.

Surgical procedure to create AV fistulae and grafts (operation to place your access)

7. **Selection of fistula or graft type** (which veins are used to create your access and how they are connected to the artery)

Possible options are: Veins that are located more at the surface of the skin *versus* deeper veins; Veins that are taken from a different part of your body than where the access is placed; Repositioning your vein before connecting it to the artery *versus* leaving it in its original position.

O	O	O	O	O
Not important	A little important	Moderately important	Important	Very important

8. **Selection of AV graft material** (the material of the tube that is used to connect your vein with an artery; this only refers to grafts)

Possible options are: Synthetic material that allows routine use for dialysis after 3-4 weeks *versus* biological material that can be routinely used after 1-2 weeks; Material that seals itself closed to shorten the time needed to stop the bleeding after removing the dialysis needle.

O	O	O	O	O
Not important	A little important	Moderately important	Important	Very important

9. **Timing of surgical procedure** (when to have your access operation)

Possible options are: Having the access operation long before your first haemodialysis session *versus* postponing it for as long as possible with the risk that you need a catheter during your first period on dialysis.

O	O	O	O	O
Not important	A little important	Moderately important	Important	Very important

10. **Local regional *versus* general anesthesia**

Possible options are: Being conscious during the access operation without feeling pain *versus* being fully unconscious.

O	O	O	O	O
Not important	A little important	Moderately important	Important	Very important

11. Prevention, diagnosis and treatment of peri-operative fistula thrombosis (preventing, diagnosing and removing blood clots in your access during and shortly after the operation)

Possible options are: (for prevention and diagnosis) Doing nothing; Administration of drugs during and shortly after the access operation to prevent your blood from clotting, or to prevent your arteries from contracting in order to ensure sufficient blood flow; Regularly checking your access yourself for a thrill (vibration), pulse or bruit (swishing sound) the days following the access operation; Examination of your vessels with ultrasound the days following the access operation; Examination of your access by inserting a needle into it and injecting contrast dye so that the blood vessels can all be seen with X-ray (angiography) in the days following the access operation; (for treatment) Injection of drugs to dissolve the clot (clot busting); Operation to remove the blood clot in your access.

O	O	O	O	O
Not important	A little important	Moderately important	Important	Very important

12. Prevention of peri-operative infections (preventing access infections during and shortly after your access operation)

Possible options are: Doing nothing; Administration of antibiotics during or after the access operation; Daily changing of the wound dressing in the days following the access operation; Inspecting the wound for signs of infection (like redness, pain or pus).

O	O	O	O	O
Not important	A little important	Moderately important	Important	Very important

Maturation of AV fistulae (the enlargement of your access after the operation before it can be used for dialysis; this only refers to fistulae)

13. Defining and assessing AV fistula maturation (checking if your fistula is growing sufficiently large)

Possible options are: Regular physical examinations of your access arm by your doctor; Examination of your access with ultrasound to determine blood flow and diameter.

O	O	O	O	O
Not important	A little important	Moderately important	Important	Very important

14. Prevention of poor AV fistula maturation (preventing insufficient enlargement of your fistula)

Possible options are: Rest until the swelling of your access arm or leg is resolved; Exercising your access hand/arm for 15-20 minutes several times a day (for example, by squeezing a rubber ball); Closing some of the other vessels in your access arm (during your access operation) to increase the blood flow through your fistula.

O	O	O	O	O
Not important	A little important	Moderately important	Important	Very important

15. Interventions for poor AV fistula maturation (treatment in case of insufficient enlargement of your fistula)

Possible options are: Inserting a needle into your access arm and inflating a special balloon to widen your fistula (angioplasty); Operation to widen your fistula; Operation to close some of the smaller vessels in your access arm (after your fistula operation) to increase the blood flow through your fistula; Operation to place a new fistula.

O	O	O	O	O
Not important	A little important	Moderately important	Important	Very important

Cannulation of AV fistulae and grafts (insertion of the dialysis needles into your access)

16. Timing of first cannulation (when should needles first be used in your access for dialysis)

Possible options are: As early as possible so that the temporary catheter can be removed or is not even necessary *versus* postpone as long as possible to be sure the fistula/graft is ready for use.

O	O	O	O	O
Not important	A little important	Moderately important	Important	Very important

17. Selection of cannula type (selecting a type of dialysis needle)

Possible options are: Different needle sizes; Using smaller needles for the first sessions before using larger ones.

O	O	O	O	O
Not important	A little important	Moderately important	Important	Very important

18. Managing pain during cannulation (dealing with pain when inserting dialysis needles)

Possible options are: Doing nothing; Pulling the skin taut to allow easier needle insertion; Applying a pain killing cream to numb the skin before needle insertion.

O	O	O	O	O
Not important	A little important	Moderately important	Important	Very important

19. Managing needle phobia (dealing with extreme needle fear)

Possible options are: Doing nothing; Tilting the dialysis chair to prevent dizziness and passing out; Psychological assistance or therapy to help you get over the fear.

O	O	O	O	O
Not important	A little important	Moderately important	Important	Very important

20. Cannulation technique (techniques for inserting the dialysis needles)

Possible options are: Applying a cuff before needle insertion to improve the visibility of the vessel *versus* not applying a cuff; Rotating sites *versus* using exactly the same site to insert the needle; Having dialysis staff inserting the needles *versus* being trained to put your dialysis needles in yourself.

O	O	O	O	O
Not important	A little important	Moderately important	Important	Very important

21. Establishing hemostasis after cannulation (to stop the bleeding after haemodialysis sessions)

Possible options are: Gently pressing your access yourself with a clean gauze pad for at least 10 minutes after removing the needle; Someone else (such as a nurse or a relative) gently pressing your access with a clean gauze pad for at least 10 minutes after removing the needle; Using clamps to apply pressure; Applying special plasters or spray to stop the bleeding more rapidly.

O	O	O	O	O
Not important	A little important	Moderately important	Important	Very important

AV fistulae and grafts complications (problems with your fistula or graft once you are on dialysis)

22. Surveillance to detect fistula or graft dysfunction (regular checks of the functioning of your access)

Possible options are: Daily checking your access yourself for a thrill (vibration), pulse or bruit (swishing sound); Regularly having someone from your care team checking the values on your haemodialysis machine; Physical examination of your access by a nurse before each dialysis session; Monthly ultrasound examinations of your access at the start of a haemodialysis session.

O	O	O	O	O
Not important	A little important	Moderately important	Important	Very important

23. Prevention, diagnosis and treatment of stenosis (preventing, diagnosing and removing a narrowing in your access)

Possible options are: (for prevention and diagnosis) Doing nothing; Regularly having someone from your care team checking the values on your haemodialysis machine; Rotating sites *versus* using exactly the same site to insert the needle; Physical examination of your access by a nurse before each dialysis session; Monthly ultrasound examinations of your access at the start of a haemodialysis session; Examination of your access by inserting a line into the access arm and injecting contrast dye so that the vessels can be seen with X-ray (angiography); (for treatment) Inserting a needle into your access arm and inflating a special balloon to open up the narrowing (angioplasty); Operation to open up the narrowing.

O	O	O	O	O
Not important	A little important	Moderately important	Important	Very important

24. Prevention, diagnosis and treatment of thrombosis (preventing, diagnosing and removing blood clots in your access)

Possible options are: (for prevention and diagnosis) Doing nothing; Tablets to prevent your blood from clotting; Treating a narrowing that increases the risk of clotting; (for treatment) Operation to remove the blood clot in the access; Operation to place a new access.

O	O	O	O	O
Not important	A little important	Moderately important	Important	Very important

25. Prevention and diagnosis of infection (preventing and diagnosing access infections)

Possible options are: Doing nothing; Washing your access site with antibacterial soap daily and before dialysis; Cleaning your access site with disinfectant after each dialysis session; Not scratching your access; Watching for signs of infection (like redness, swelling, pain or pus).

AV fistulae and grafts adverse effects (unwanted consequences of having a fistula or graft)

26. Prevention, diagnosis and treatment of perigraft seroma (preventing, diagnosing and removing a (sometimes painful) swelling around your graft)

Possible options are: (for prevention and diagnosis) Doing nothing; Examination of your graft with ultrasound; (for treatment) Using a syringe to take out fluid from the swelling; An operation to remove the swelling; Operation to place a new graft.

O	O	O	O	O
Not important	A little important	Moderately important	Important	Very important

27. Prevention and treatment of AV access related heart disease (preventing and treating heart disease caused by having a fistula or graft)

Possible options are: Doing nothing; Operation to reduce the size of your fistula; Operation to close or remove the access.

O	O	O	O	O
Not important	A little important	Moderately important	Important	Very important

28. Diagnosis and treatment in case of atypical arm/hand pain (diagnosing and treating an unexplained pain in your access arm/hand)

Possible options are: (for diagnosis) Doing nothing; Physical examination of your access arm/hand by your doctor; Examination of your access with ultrasound; (for treatment) Doing nothing; Taking pain killers.

O	O	O	O	O
Not important	A little important	Moderately important	Important	Very important

29. Prevention and treatment of limb ischemia (preventing and treating insufficient blood supply to your arms or legs)

Possible options are: (for prevention) Doing nothing; Watching out for cold or blue fingers/toes or sores at the tips of your fingers/toes; Tablets to thin your blood; Quit smoking; (for treatment) Operation to close or remove the access; Bypass operation to restore the blood flow; Operation to remove the part of the arm or leg that has died off due to insufficient blood supply.

O	O	O	O	O
Not important	A little important	Moderately important	Important	Very important

30. Prevention and treatment of aneurysm (preventing and treating bulging of the wall of your access)

Possible options are: (for prevention) Doing nothing; Periodically checking your access for narrowing or signs of your access's wall bulging; Rotating sites *versus* using exactly the same site to insert the needle; (for treatment) Doing nothing; Corrective operation on your access; Operation to place a new access in another site if available.

O	O	O	O	O
Not important	A little important	Moderately important	Important	Very important

31. Prevention and treatment of carpal tunnel syndrome (preventing and treating a disorder of the wrist and hand which includes numbness and pain)

Possible options are: (for prevention) Avoiding repetitive movements (like mouse clicking, knitting); Adjusting your work and life environment; (for treatment) Wearing a wrist brace; Anti-inflammatory drugs; Hormone injections; An operation.

O	O	O	O	O
Not important	A little important	Moderately important	Important	Very important

---

Your care when placing and having a vascular access – Tunneled catheters

The topics in this section all concern aspects of the operation to place a tunneled catheter as your vascular access, and the care you receive to prevent and treat problems with your catheter once you are on dialysis.

Please rate the importance of each of the following topics

For each topic, assume that all options are equally safe and effective and then ask yourself: *'How strongly do I prefer one option over the others?'*, and *'How much evidence would my doctor need to convince me that the other options are better?'*.

Catheter insertion (placement of your catheter)

32. Selection of catheter type and material (selecting the type of catheter tube)

Possible options are: Size of the catheter tube (diameter and length); Stiffness of the catheter tube; Covering the inner catheter wall with a special coating *versus* not using a coating.

O	O	O	O	O
Not important	A little important	Moderately important	Important	Very important

33. Insertion methods (how your catheter is inserted)

Possible options are: Ultrasound to guide the insertion of your catheter (which prolongs the procedure) *versus* anatomical features to guide catheter insertion; Being conscious during your access operation without feeling pain *versus* being fully unconscious.

O	O	O	O	O
Not important	A little important	Moderately important	Important	Very important

34. Verification of catheter tip position (checking the position of your catheter)

Possible options are: Doing nothing; Using X-ray to verify the position of your catheter after insertion.

O	O	O	O	O
Not important	A little important	Moderately important	Important	Very important

Catheter complications and adverse effects (problems with your catheter once you are on dialysis)

35. Surveillance to detect catheter dysfunction (regular checks of the functioning of your catheter)

Possible options are: Doing nothing; Regularly having someone from your care team checking the values on your haemodialysis machine.

O	O	O	O	O
Not important	A little important	Moderately important	Important	Very important

36. Prevention and treatment of thrombosis (preventing and removing blood clots in your catheter)

Possible options are: (for prevention) Doing nothing; Tablets to prevent your blood from clotting; Not allowing anyone to use your catheter for things other than dialysis (such as drawing blood or injecting medication); Injecting an anti-clotting fluid directly into your catheter after each dialysis session, which locks it until the next session; flushing your catheter with a saline solution before locking it; (for treatment) Injecting blood thinning medication directly into your catheter; Having an infusion lasting several hours to unblock your catheter; Replacing your clotted catheter by a new one.

O	O	O	O	O
Not important	A little important	Moderately important	Important	Very important

37. Prevention and diagnosis of catheter infections (preventing and diagnosing catheter infections)

Possible options are: Doing nothing; Not taking showers; Special dressings that can be left in place for 1-2 weeks and allow regular checking of your catheter without having the dressings removed; Allowing only dialysis nurses to remove/change your dressings; Injecting an antibacterial fluid directly into your catheter after each dialysis session, which locks it until the next session; Watching for signs of infection (like redness or pus near the exit site, fever).

O	O	O	O	O
Not important	A little important	Moderately important	Important	Very important

38. Prevention and treatment of central vein obstruction (preventing and treating a narrowing of the central vein in which your catheter is placed)

Possible options are: (for prevention) Doing nothing; Minimizing the time you need a catheter; Placing your catheter in a vessel in which a narrowing is least likely to occur; (for treatment) Doing nothing; Inserting a needle into the vessel with the narrowing (angioplasty) and inflate a special balloon to open up the narrowing.

O	O	O	O	O
Not important	A little important	Moderately important	Important	Very important

How vascular access is organised in your centre

The three topics in this last section concern aspects related to how your centre organises vascular access care in general.

Please rate the importance of each of the following topics

For each topic, assume that all options are equally safe and effective and then ask yourself: *'How strongly do I prefer one option over the others?'*, and *'How much evidence would my doctor need to convince me that the other options are better?'*.

39. Training and education of clinicians in creation and maintenance (training of your dialysis team in how to place and take care of your vascular access)

Possible options are: Kidney doctors and nurses in your centre learning all about vascular access care during their basic training *versus* during special courses in addition to their basic training; Having fistula/graft operations done only by surgeons who undertook special training for fistula/graft placement; Having fistula/graft operations done only by surgeons who placed at least 30 fistulae in the last year.

O	O	O	O	O
Not important	A little important	Moderately important	Important	Very important

40. Strategies to organise vascular access care (the way your dialysis centre organises the care you need during and after a vascular access procedure)

Possible options are: Having your vascular access care coordinated primarily by your kidney doctor *versus* by a team of clinicians (kidney doctor, renal nurses, surgeon); Having your fistula/graft placed by a surgeon in the nearest hospital *versus* in a hospital further away that is specialised in placing fistulae; Taking part in training to learn what you can do yourself regarding your vascular access care.

O	O	O	O	O
Not important	A little important	Moderately important	Important	Very important

41. **Involvement of patient and carers in access maintenance** (the support you and your carers get for playing an active role in taking care of your access)

Possible options are: Taking part in training to learn what you can do yourself regarding your vascular access care; Folders with information on how to protect your fistula from damaging.

O	O	O	O	O
Not important	A little important	Moderately important	Important	Very important

42. Monitoring the quality of vascular access care (regular checks of the overall quality of the vascular access care your dialysis centres provides)

Possible options are: Your dialysis team having monthly meetings to discuss if the vascular access care in the centre is of sufficient quality; Your dialysis team analysing all cases of catheter infection in the centre to identify potential reasons for the infection; An external organisation annually visiting your dialysis centre to check if the centre's vascular access care is of sufficient quality.

O	O	O	O	O
Not important	A little important	Moderately important	Important	Very important

Suggestion for additional topics

Do you feel there are any aspects of vascular access care that should be covered by the new guideline, but were not in the list?

Suggestion 1: .....[free text]

Suggestion 2: .....[free text]

Suggestion 3: .....[free text]
