# Supplementary material for: Setting Priorities for Optimizing Vascular Access Decision Making – An International Survey of Patients and Clinicians
Source: PLoS One. 2015 Jul 7;10(7):e0128228. doi: 10.1371/journal.pone.0128228 (PMC4494812; doi:10.1371/journal.pone.0128228)
Supplement: S3 File — Results are reported per respondent group. (PDF) [file pone.0128228.s003.pdf]

S3 File – Mean adjusted ratings and ranks of all 42 vascular access topics included in the survey (reported per respondent group)

		**KIDNEY PATIENTS**			**CLINICIANS**								
					Nephrologists			*Nurses*			*Surgeons and radiologists*	
**Topic**	**N**	**Mean (SD) rating$_{adj}$**	**Rank**	**N**	**Mean (SD) rating$_{adj}$** [a)]	**Rank**	**N**	**Mean (SD) rating$_{adj}$** [a)]	**Rank**	**N**	**Mean (SD) rating$_{adj}$** [a)]	**Rank**
**Preservation of veins**	84	4.24 (0.51)	18	687	**4.45 (0.36)**	3	194	**4.43 (0.33)**	2	140	**4.56 (0.34)**	2
**Timing of vascular access creation** (e.g., when to start talking about it with the patient, when to first assess the vessels, time of surgical referral)	84	4.21 (0.39)	26	687	**4.39 (0.35)**	7	194	4.24 (0.36)	22	140	**4.38 (0.33)**	9
**Preoperative assessment of vessels** (e.g., general clinical evaluation, imaging techniques for vein mapping, cut off values for suitable vessels)	85	4.33 (0.40)	7	687	4.30 (0.38)	18	194	4.20 (0.41)	24	140	**4.49 (0.34)**	5
**Patient involvement in preparing for access creation and type selection** (e.g., educational strategies, decision-making aids, psychosocial support)	85	4.25 (0.43)	17	687	**4.05 (0.46)**	32	194	4.17 (0.41) [b)]	28	140	4.13 (0.43)	27
**Selection of vascular access type** (e.g., tunneled catheter versus AV fistula versus AV graft, clinical and social (contra-)indications for specific access types, last resort access types)	85	4.39 (0.29)	2	687	**4.49 (0.31)**	2	194	4.34 (0.37)	9	140	**4.57 (0.24)**	1
**Selection of vascular access site** (e.g., upper versus lower limb for fistulas/grafts, internal jugular versus subclavian vein for catheters)	85	4.32 (0.36)	9	687	4.40 (0.35)	5	194	4.32 (0.36)	15	140	**4.50 (0.27)**	4
**Surgical techniques for AV access creation** (e.g., selection of fistula or graft type, microsurgery, use of vessel dilators)	75	4.10 (0.43)	33	687	**4.39 (0.35)**	8	194	4.24 (0.43)	21	140	**4.54 (0.28)**	3
**Selection of AV graft material** (e.g., synthetic versus autologous, hybrid grafts)	68	3.93 (0.59)	38	687	3.88 (0.51)	37	194	3.93 (0.55)	37	140	**4.21 (0.54)**	22
**Timing of surgical procedure to create AV access** (i.e., when to create the access)	76	4.09 (0.54)	35	687	**4.29 (0.39)**	19	194	4.10 (0.44)	33	140	4.26 (0.36) [b)]	20
**Local regional versus general anesthesia**	75	4.07 (0.49)	36	687	**3.58 (0.53)**	41	194	**3.54 (0.59)**	41	140	**3.76 (0.54)**	40
**Prevention, diagnosis & treatment of peri-operative fistula thrombosis** (i.e., during and shortly after surgery)	75	4.30 (0.37)	12	687	4.26 (0.36)	20	194	4.56 (0.28)	6	140	4.07 (0.40)	21
**Prevention of peri-operative fistula/graft infections**	76	4.34 (0.34)	5	687	4.27 (0.41)	22	194	4.40 (0.33)	4	140	**4.20 (0.46)**	23
**Defining & assessing AV fistula maturation**	76	4.29 (0.33)	13	687	4.34 (0.33)	11	194	4.33 (0.33)	12	140	4.31 (0.33)	15
**Prevention of poor AV fistula maturation** (e.g., indications for preemptive interventions, forearm or	75	4.19 (0.33)	28	687	**4.32 (0.36)**	16	194	**4.32 (0.31)**	13	140	4.28 (0.34)	19

upper limb exercise, obliteration of venous branches)															
**Interventions for poor AV fistula maturation** (e.g., surgery, angiography)	74	4.25 (0.37)	16		687	4.30 (0.38)	17		194	4.20 (0.41)	25		140	4.33 (0.38)	13
**Timing of first cannulation**	76	4.17 (0.38)	30		687	4.22 (0.38)	24		194	4.25 (0.39)	20		140	4.14 (0.37)	25
**Selection of cannula type** (e.g., needle gauge, needle material, using smaller needles for first cannulation)	75	4.01 (0.48)	37		687	3.98 (0.44)	35		194	4.10 (0.42)	32		140	3.89 (0.35)	36
**Managing pain during cannulation**	76	3.90 (0.65)	40		687	3.79 (0.44)	39		194	4.06 (0.44)	34		140	3.81 (0.39)	38
**Managing needle phobia**	76	3.69 (0.57)	42		687	3.76 (0.47)	40		194	**3.99 (0.47)**	35		140	3.77 (0.43)	39
**Cannulation procedure** (e.g., button hole versus rope ladder technique, needle orientation, use of imaging techniques, self-cannulation, avoiding needle dislodgment during dialysis, tourniquet use, needling of deep fistulas, puncture site surveillance)	75	4.22 (0.48)	25		687	4.13 (0.45)	28		194	4.27 (0.39)	18		140	4.11 (0.37)	29
**Establishing hemostasis after cannulation** (e.g., use of hemostatic plaster, use of clamps, management of malpuncture incidents)	76	4.11 (0.48)	34		687	4.13 (0.40)	27		194	4.19 (0.42)	27		140	4.08 (0.38)	30
**Surveillance of fistula/graft (dys)function** (e.g., methods, frequency, cut-off values for blood flow/venous pressure/recirculation)	76	4.26 (0.30)	14		687	4.34 (0.35)	12		194	4.33 (0.34)	10		140	4.33 (0.35)	12
**Prevention, diagnosis & treatment of fistula/graft stenosis** (e.g., preemptive invasive interventions, interventional radiology, timing of interventions)	76	4.23 (0.35)	20		687	**4.37 (0.34)**	9		194	**4.36 (0.29)**	8		140	**4.38 (0.34)**	10
**Prevention, diagnosis & treatment of fistula/graft thrombosis** (e.g., oral anticoagulants, effect of medication (ESAs, statins) on thrombosis risk, preemptive invasive interventions, thrombectomy, timing of interventions)	76	4.31 (0.34)	11		687	4.36 (0.34)	10		194	4.40 (0.27)	5		140	4.37 (0.35)	11
**Prevention &diagnosis of fistula/graft infection** (e.g., aseptic techniques before cannulation, prophylactic antibiotics)	76	4.35 (0.36)	6		687	4.40 (0.33)	4		194	4.41 (0.29)	3		140	4.31 (0.32)	16
**Prevention, diagnosis &treatment of perigraft seromas** [c)]	46	4.25 (0.38)	19		268	**3.54 (0.61)**	42		87	**3.53 (0.75)**	42		70	**3.58 (0.55)**	42
**Prevention &treatment of AV access related heart disease**	71	4.33 (0.40)	8		687	**4.11 (0.41)**	29		194	**4.15 (0.37)**	30		140	**3.99 (0.40)**	35
**Diagnosis & treatment in case of atypical arm/hand pain** [c)]	46	4.26 (0.42)	15		268	**4.01 (0.49)**	33		87	**3.83 (0.61)**	40		70	4.08 (0.55)	31
**Prevention &treatment of limb ischemia** (e.g., diagnosis/treatment of steal syndrome)	74	4.24 (0.39)	21		687	4.32 (0.34)	15		194	4.19 (0.36)	26		140	4.32 (0.31)	14
**Prevention &treatment of aneurysms**	76	4.16 (0.41)	31		687	4.10 (0.37)	30		194	4.15 (0.40)	29		140	4.13 (0.37)	26

**Prevention &treatment of carpal tunnel syndrome**	74	3.90 (0.44)	39		687	3.84 (0.42)	38		194	3.94 (0.46)	36		140	**3.70 (0.49)**	41
**Selection of catheter type, material, and site** (e.g., coating, antegrade/retrograde tunneling, single/double lumen)	9	4.14 (0.56)	32		687	3.99 (0.43)	34		194	3.85 (0.51)	39		140	3.89 (0.42)	37
**Catheter insertion methods** (e.g., ultrasound guided versus anatomic landmarks)	9	3.82 (0.52)	41		687	4.08 (0.48)	31		194	3.91 (0.54)	38		140	4.07 (0.45)	32
**Verification of catheter tip position**	9	4.23 (0.37)	22		687	4.22 (0.41)	25		194	4.28 (0.38)	17		140	4.15 (0.42)	24
**Surveillance of catheter (dys)function** (e.g., methods, frequency, cut-off values for blood flow, long-term surveillance)	9	4.20 (0.28)	27		687	4.18 (0.38)	26		194	4.25 (0.38)	19		140	4.00 (0.40)	33
**Prevention, diagnosis & treatment of catheter thrombosis** (e.g., anticoagulants, lock solutions, catheter exchange, timing of interventions)	9	4.47 (0.33)	1		687	4.33 (0.33)	14		194	4.33 (0.33)	11		140	4.12 (0.37) [b)]	28
**Prevention & diagnosis of catheter infections** (e.g., (dis)connection techniques, exit site care, taking showers, lock solutions, (timing of) catheter exchange)	9	4.36 (0.19)	4		687	4.53 (0.27)	1		194	4.49 (0.27)	1		140	4.30 (0.37)	18
**Prevention and treatment of central vein obstruction**	9	4.22 (0.26)	24		687	4.40 (0.31)	6		194	4.36 (0.29)	7		140	4.41 (0.32)	7
**Training, certification & monitoring of skills/expertise of health care professionals in creation and maintenance** (e.g., who should be trained for what, educational strategies, minimum requirements for number of procedures per surgeon)	85	4.37 (0.41)	3		687	4.34 (0.34)	13		194	4.32 (0.34)	14		140	4.41 (0.36)	8
**Strategies to organize vascular access care** (e.g., who should create fistulas, multidisciplinary teams, vascular access coordinators, care pathways, dedicated surgical resources, specialized vascular access centres)	85	4.31 (0.37)	10		687	4.28 (0.37)	21		194	4.31 (0.35)	16		140	4.42 (0.34)	6
**Involvement of patient and family in access maintenance** [c)] (e.g., promoting self-management, educational programmes, decision-making aids)	50	4.23 (0.44)	23		268	**3.98 (0.56)**	36		87	4.10 (0.59)	31		70	4.00 (0.56)	34
**Monitoring & improving quality of vascular access care** (e.g., performance feedback, quality audits, financial incentives, meaningful performance indicators)	85	4.20 (0.38)	29		687	4.22 (0.38)	23		194	4.22 (0.42)	23		140	4.31 (0.36)	17

Abbreviations: AV, arteriovenous; N, number of respondents who rated the importance of a topic; SD, standard deviation

a) Values in bold indicate that the rating significantly differed from the rating assigned by patients ($P < 0.01$).

b) Difference with patient rating was borderline significant (P between 0.010 and 0.014)

c) Topic was included in the survey based on suggestions by clinicians and patients in Phase 1. Data on rating were collected in Phase 2.
